# Phenotypic drug resistance and genetic mutations linked to resistance among extrapulmonary tuberculosis patients in Ethiopia: Insights from Whole Genome Sequencing

**DOI:** 10.21203/rs.3.rs-5302564/v1

**Published:** 2024-12-17

**Authors:** Hilina Mollalign, Dawit Hailu Alemayehu, Dereje Beyene, Kalkidan Melaku, Abaysew Ayele, Dawit Chala, Getu Diriba, Bazezew Yenew, Muluwork Getahun, Bethlehem Adnew, Shewki Moga, Jeffrey Michael Collins, Arash Ghodousi, Kidist Bobosha, Liya Wassie

**Affiliations:** Ethiopian Public Health Institute; Armauer Hansen Research Institute; Addis Ababa University; Armauer Hansen Research Institute; Armauer Hansen Research Institute; Ethiopian Public Health Institute; Ethiopian Public Health Institute; Ethiopian Public Health Institute; Ethiopian Public Health Institute; Armauer Hansen Research Institute; Ethiopian Public Health Institute; Emory University; Vita-Salute San Raffaele University; Armauer Hansen Research Institute; Armauer Hansen Research Institute

**Keywords:** Extrapulmonary tuberculosis, Whole genome sequencing, Ethiopia

## Abstract

Globally, drug-resistant tuberculosis (DR-TB) is responsible for 13% of mortality attributable to antimicrobial resistance. In Ethiopia, extrapulmonary tuberculosis (EPTB) is a significant public health challenge, and drug resistance (DR) in EPTB is often overlooked. In a cross-sectional study conducted between August 2022 and October 2023, we aimed to explore the magnitude of phenotypic drug resistance and identify genetic mutations linked to resistance using 189 Mycobacterium tuberculosis (MTB) isolates cultured from extrapulmonary clinical specimens. Additionally, we assessed the agreement of the phenotypic and whole genome sequencing (WGS) based genotypic drug resistance detection. We performed phenotypic drug sensitivity testing (pDST) using liquid culture BD BACTECTM MGITTM 960 system and WGS using Illumina NextSeq500/550. The genomic data analysis pipelines MTBSeq and TBProfiler were used to predict drug resistance-conferring mutations. The agreement between the pDST and WGS was analyzed using SPSS version 29.0 software. Our result demonstrated phenotypic resistance to at least one anti-TB drug was detected in 16.9% (32/189) of the study participants. Isoniazid-resistant rifampicin-susceptible-TB (Hr-TB) and multi-drug-resistant TB (MDR-TB) phenotypes accounted for 2.6% (5/189) and 4.2% (8/189) respectively. Prevalence of MDR-TB was 2.4% (4/170) among newly diagnosed and 21.1% (4/19) among previously treated cases. WGS identified more (14/160, 8.75%) rifampicin-resistant genotypes (RR-TB) compared to pDST (8/189, 4.2%). We have identified a putative compensatory mutation for rifampicin (rpoBSer450Leu, rpoCAsp747Ala) for the first time from an EPTB clinical specimen in Ethiopia. Overall, there was a 3.75% rifampicin mono-resistant-TB(RMR-TB) genotype, which remains undetected using the conventional pDST and represented 42.9% (6/14) of the identified RR-TB genotypes. Mutations conferring rifampicin resistance-interim (rpoB.Ser450Ala) represented the majority (83.3%) of RMR-TB. Changes in ethA genes associated with ethionamide resistance were the most common resistance (n=7, 87.5%) in MDR-TB cases. There was a strong agreement between the pDST and WGS-TB Profiler pipeline to detect RR-TB (kappa=0.8) compared to the MTBSeq pipeline (k=0.58). In conclusion, MDR-TB, Hr-TB, and interim-RMR-TB are equally important public health challenges in the realm of EPTB in Ethiopia. The role of WGS is tremendous in detecting borderline/interim RMR-TB, which will help for tailored, personalized treatment strategies.

## Introduction

Tuberculosis (TB) is a leading cause of morbidity and mortality globally where in 2022, an estimated 10.6 million people fell ill with TB, and more than 1.3 million died^1^. The continued emergence of drug-resistant TB (DR-TB) remains a major hurdle to the global TB control efforts. Globally, 13% of mortality attributable to antimicrobial resistance is due to DR-TB^2^. In 2022, an estimated 410,000 multidrug resistant TB (MDR-TB) cases were diagnosed, and only two in five infected persons began treatment, with only 63% treatment success rate^1^.

Ethiopia is among the high TB/TB-HIV burden countries^3^, with an annual TB incidence of 126 per 100,000 population^1^. Extrapulmonary TB (EPTB), defined as a TB disease affecting any organ system other than the lung, has remained a public health challenge in Ethiopia, representing 29–31% of the notified TB cases over the last decade^1,4,5^. TB lymphadenitis is the most predominant (80%) form of EPTB, and the contributing factors related to its high prevalence are not yet fully explained^6^. Hence, determinants of EPTB have been outlined as a top research priority in the national TB research road map^5^. Ultrasound-guided cytology is the most widely used diagnostic method, and poor treatment outcome remains a major concern^5^. Baseline or follow-up drug sensitivity test (DST) is often overlooked to guide treatment, because clinical specimens from EPTB patients are often pauci-bacillary and involve invasive sample collection procedures. Thus, treatment options are often extrapolated from pulmonary TB (PTB), and there is a paucity of information about drug resistances and genetic mutation profiles linked to resistance in EPTB.

Genotypic drug sensitivity test (gDST) can potentially underpin the phenotypic drug sensitivity test (pDST) due to the strong agreement and with rapid detection of resistance to rifampicin (RIF) and isoniazid (INH)^7^. However, discordances have been also reported for the detection of other anti TB regimens^7,8^. Whole genome sequencing (WGS), an advanced genotyping method, can predict drug resistances including resistances occurring outside the hotspot resistance determining regions. Furthermore, WGS can detect mutations associated with borderline and interim resistances that remain undetected using the phenotypic and other conventional molecular methods. Thus WGS-based resistance detection in different settings assist to develop and/or update the rapid molecular assays for detecting resistances in clinical specimens^1^. Furthermore, it could also help to complete the World Health Organization’s (WHO) mutation catalogue of *Mycobacterium tuberculosis* (MTB)^9^.

Most clinical *Mycobacterium tuberculosis* (MTB) examined for drug resistance prediction using WGS have been isolated from patients with PTB^7,10^. Despite the significant contribution of EPTB to the global and national TB case load, WGS data from these patients and their association with phenotypic resistance are not well established. In this study, we aimed to explore phenotypic drug resistance in MTB isolates from EPTB patients and identify genetic mutations linked to resistance using WGS. Additionally, we assessed the agreement between the pDST and WGS.

## Results

### Characteristics of study participants

MTB isolates from 189 study participants were tested for phenotypic drug resistance. The overall mean age of the study participants was 32 years (95% CI: 30–34 years). More than half (102/189, 54%) of the study participants were males. Contact history to active TB index case was reported by 22.8%, while the remaining either had no known contact history (66.7%) or were uncertain to report about their contact history (10.5%). Previous treatment history was reported only by 10.1%, while the remaining 89.9% were newly diagnosed at the time of enrollment. Previous treatment history was associated with drug resistance (p<0.001). Table 1 describes participants’ characteristics across drug sensitivity profile.

### First and second line phenotypic drug resistance detection

Phenotypic resistance to at least one anti-TB drug was detected in 16.9% (32/189) isolates. Isoniazid resistant-rifampicin susceptible TB (Hr-TB) and MDR-TB/RR-TB phenotypes accounted for 2.6% (5/189) and 4.2% (8/189), respectively. Prevalence of MDR-TB among newly diagnosed and previously treated cases were 2.4% (4/170) and 21.1% (4/19), respectively, whereas Hr-TB accounted for 1.8% (3/170) and 10.5% (2/19) in newly diagnosed and previously treated cases, respectively. There was a statistically significant association between previous TB treatment history and drug resistance (*p<0.001*). Resistance to delamanid was detected in one (0.5%) MDR-TB patient and WGS identified a missense variant (fbiC. Ser706Pro) at 18.31% allele frequency. However, this mutation has not been described in the catalogue of mutations^9^. Resistance to second line injectables, capreomycin (3.7%) and kanamycin (2.7%) were detected mostly in non MDR -TB cases, but no resistance to amikacin was detected. Only one MDR-TB and one Hr-TB isolates showed resistance to capreomycin. Table 2 shows the proportion of resistant isolates detected for each anti-TB drug.

### WGS based drug resistance detection

**Figure 1:**
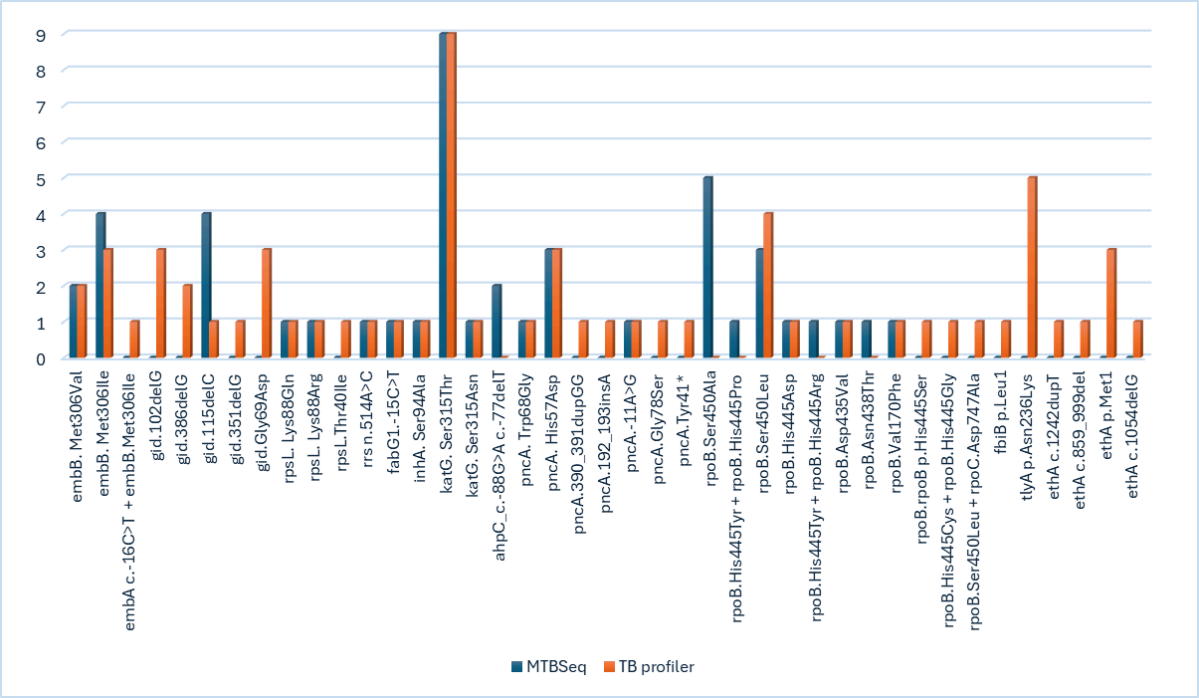
Frequency of resistance conferring mutations detected by TB profiler and MTBSeq among EPTB isolates

Whole genome sequencing-based drug resistance results were available for 160 isolates. The frequency of genetic drug resistance conferring mutations as depicted by TB profiler and MTBSeq for each drug is presented in Figure 1. WGS detected 87.5% of phenotypically identified MDR-TB. Phenotypic DST, on the other hand, failed to detect one MDR-TB genotype, which has a missense variant (rpoB.His445Ser). This mutation is a group-2 WHO confidence grade mutation associated with borderline rifampicin resistance^9^.

MTBSeq detected more rifampicin resistant-TB (RR-TB) cases; 8.75% (n=14/160) than TB Profiler; 5% (n=8/160) and pDST; 4.2% (8/189). Nearly half (6/14, 42.9%) of these RR-TB appeared interim rifampicin mono resistant-TB (RMR-TB) (Table 3). WGS detected MDR-TB in 5%, all with high-level isoniazid (INH) resistance mutation (KatG.Ser315Thr). Moreover, one MDR-TB isolate had a putative compensatory mutation for rifampicin (rpoBSer450Leu, rpoCAsp747Ala). This MDR-TB isolate had a double point mutation and one phylogenetic single nucleotide polymorphism (SNP) at INH resistance determining region (inhASer94Ala, katGSer140Asn phylo SNP, katGSer315Asn). This isolate was identified from a male, 19-year-old TB retreatment patient from a rural part of Ethiopia. The patient has reported a history of contact with an active TB case. The patient was a TB treatment defaulter with a history of treatment interruption for more than two months. A putative compensatory mutation is defined based on a previously reported criteria^21^ in which the isolate should carry a secondary RNA polymerase mutation (rpoC/A mutation) with a primary mutation on hotspot rifampicin resistance determining region (RRDR), mostly rpoBSer450Leu, the slow growth rate of the primary culture and the mutation shall never happen in drug susceptible isolate. Based on this, our study isolate harbors a mutation that has been reported as a compensatory mutation^22–24^. To our knowledge, this is the first compensatory mutation report from Ethiopia identified from clinical EPTB specimen. Furthermore, an upstream gene variant of (ahpC_c.−88G>A c.−77delT) was detected among MDR-TB and Hr-TB carrying KatG mutations. A mutation on ahpC gene is known to compensate for the katG deficit of isoniazid resistance^25^.

### Hetero-resistance detection and prevalence

Hetero resistance, which is a precursor to the development of fully resistant populations, was identified based on the frequency of alleles for a specific variant using WGS. In this study hetero resistance was detected in RMR-TB only. At a 10% threshold (4/6, 66.7%) and at a 5% threshold, two (2/6, 33.3%) of the identified RMR-TB showed hetero-resistance (Table 3). In these genotypes, variants conferring drug resistance appeared at an average allele frequency of 12.6%.

### Level of agreement between WGS and pDST

A comparative analysis of MTBSeq and TBProfiler pipelines using the phenotypic method as a reference showed that both pipelines detected 76.9% of pINH resistances with 23.1% phenotype-genotype discordances. Of the RR-TB phenotypes, both pipelines detected 87.5% with 12.5% discordance. MTBSeq identified six other additional RR-TB genotypes that have not been detected by the phenotypic method. There was a strong agreement between the pDST and WGS-TBProfiler pipeline for detecting RR-TB cases, kappa coefficient (*k=0.8*) as compared to MTBSeq pipeline (*k=0.58*) (Table 4). A start lost codon on fbiB gene conferring resistance for delamanid /protionamide was detected by TBProfiler in a single non-MDR-TB isolate. Polydrug resistance in MDR-TB cases was detected for ethambutol (EMB, 75%), streptomycin (STR, 100%), pyrazinamide (PZA, 75%) and ethionamide (ETO, 75%) using TBProfiler. MTBSeq, on the other hand, detected more EMB (87.5%) than streptomycin (50%) and pyrazinamide (37.5%) among MDR-TB cases.

Assessment of genomic variants in phenotypic resistant and WGS susceptible isolatesPhenotypic resistances that are not detected by WGS are summarized in Table 5. In these phenotypes, the group 3 mutations of “uncertain significance” according to the WHO mutation catalogue^9^ were identified. Manual correlation of these phenotypes with additional variants with genotypic frameshift and upstream gene variants increases the concordance of the two methods by 3 isolates for STR and PZA, where most pDST-WGS discrepancies were observed.

### Evaluation of resistance conferring mutations in susceptible phenotypes

All rpoB mutations conferring RIF resistance detected in susceptible phenotypes listed below were associated with interim genotypic resistance. The identified mutations in KatG and embB for INH and EMB respectively had also appeared in the hotspot region with no hetero resistance in these regions (Table 6).

## Discussion

In this study, we have described the phenotypic drug sensitivity and genetic mutations conferring drug resistance in MTB isolates collected from EPTB patients in Ethiopia. To our knowledge, this study is the first to report phenotypic and WGS-based genotypic data on large numbers (n=189 and 160 isolates, respectively) of EPTB clinical specimens collected from different regions of Ethiopia. In this study, there was a high rate (3.75%) of RMR-TB overall which remains undetected using the conventional DST approach. All were detected among newly diagnosed people with TB and without reported TB contact history and were thus classified as primary RMR-TB. This figure accounts for nearly half (42.8%) of RMR among RR-TB/MDR-TB cases in our study population. Mutations at rpoBSer450Ala were detected in 83.3% of these RMR-TB cases and are classified as the group-2 rifampicin resistance associated-interim in the WHO mutation catalogue^9^. These mutations appeared as a minority variant of rifampicin hetero-resistance-interim, and hence usually result in phenotypic susceptibility and poor treatment outcome^26^. Similarly a study from South Africa reported a high rate (22.7%) of RMR-TB among routinely diagnosed MDR/RR-TB patients^27^. A study from Antwerp reference laboratory also identified a borderline rpoB mutation in a proportion of 20%−30% RMR-TB among new cases of random drug resistance surveys (DRS)^28^. Such interim resistances are missed on the conventional pDST and the rapid molecular diagnostics, which have a direct clinical impact such as underdiagnosis, inadequate treatment and re-occurrence of secondary cases^29^. Given the high occurrence of such phenotypically undetected interim primary RMR in our study population, we conclude that the presence of minority variants of interim RMR-TB could contribute to poor treatment outcomes or re-occurrence of EPTB in the study setting.

It has been well understood that resistance-associated mutations result in high fitness cost, slowing the *in vitro* growth rates and transmission compared to the susceptible phenotypes^30^. Despite the increased fitness cost due to resistance mutations, rifampicin resistant phenotypes continued spreading globally, challenging the TB control effort. The potential spreading capability regardless of fitness cost in these resistant phenotypes was explained by the presence of a compensatory mutation^31^. We have reported an MDR-TB isolate carrying a compensatory mutation in the rpoC gene (rpoBSer450Leu, rpoCAsp747Ala) as defined previously^21,32^. After an intensive literature search, we report this mutation as the fourth of its type next to the first report by Casali et al followed by two isolates, reported by Alame et.al and Liu et al^22–24^. A study from South Africa explored the clinical significance of compensatory mutations and reported that most compensatory evolution in MTB was associated with smear positive PTB, increased transmission and increased mutational burden^33^. In line with this, the clinical strain of (ETB-162) had also carried multiple mutations conferring resistances to INH (inhA p. Ser94Ala katG p. Ser140Asn katG p. Ser315Asn), EMB (embB p. Met06Val), PZA (pncA p. Trp68Gly), injectable drugs (rrs n.514A>C), and ETO (ethA c.1054delG), in addition to the identified RIF resistance conferring mutations. Furthermore, the patient with this strain has a history of treatment default and reported contact history to active TB index. The clinical specimen (a 2×2cm mass lesion with actively draining sinus tracts) of this patient was initially diagnosed as acute suppurative inflammation at the time of enrollment into this study. The primary culture also showed a slow growth rate (18 days and 6 hrs for 253 growth units). The cost of slow growth rate could explain the multiple resistance-associated mutations^32^. Most compensatory mutations occurring in rpoABC genes were documented to decrease the fitness cost of rpoBSer450Leu mutation^32^.

Isoniazid, another potent first-line anti-TB drug used in the treatment of DS-TB, potentiates the effect of rifampicin and also prevents mycolic acid synthesis^34^. Because the Xpert MTB/RIF (Cepheid, Sunnyvale, CA, USA) assay only detects rifampicin resistance, and the use of Xpert XDR is recommended when rifampicin resistance is identified, diagnosis of Hr-TB has been overlooked. In this study, Hr-TB was observed in 1.8% of newly diagnosed and 10.5% of previously treated EPTB cases. Globally, Hr-TB is estimated to occur in 8% of all forms of TB^35^. Another multicountry analysis of cross-sectional data reported Hr-TB prevalence of 7.4% among new and 11.4% among previously treated patients^36^. In this study, RMR-TB is mainly identified in newly diagnosed EPTB cases whereas the proportion of Hr-TB is higher in previously treated EPTB patients. A similar finding has also been reported from PTB patients^37^.

Resistance to isoniazid also comes with increased fitness cost. To overcome this resistance-associated fitness cost, MTB restores the loss of function through co-evolution of compensatory mutation^25^. Our WGS gDST identified a compensatory mutation at the locus of the ahpC gene (ahpC_c.−88G>A c.−77delT) for a katG deficit (katGSer315Thr). Similar mutations at the oxyR-ahpC (−88g>a) intergenic region were reported among majority of Hr-TB and MDR-TB isolates^38^. Another study has also reported deletion of the upstream gene locus (−77del) as a novel compensatory effects marker with strong evidence for convergent evolution, co-occurrence with loss of function mutations in katG as well as association with INH resistant isolates^25^. Based on this evidence, our study identified KatG deficit (ahpC_c.−88G>A c.−77delT) co-occurring with katG Ser315Thr in one MDR-TB and one Hr-TB case.

Patients with Hr-TB are at an increased risk of developing MDR-TB^39^. Similarly, 90% of rifampicin resistant TB are also resistant to isoniazid, thus MDR-TB^40^. Resistance to either of these drugs results in unsuccessful treatment outcome and fuels acquisition of resistance to the other^36^. In our study, we have reported a high prevalence (4.2%) of MDR-TB which accounts for 2.4% and 21.1% among newly diagnosed and previously treated cases, respectively. In 2022, the global MDR/RR-TB prevalence was 3.3% among new and 17% among previously treated cases^1^. Though drug resistances in EPTB in our setting have not been explicitly addressed using WGS, previous studies have also reported a higher rate of MDR-TB in EPTB^41^.

The prevalence of hetero-resistance among RMR-TB was 42.9%. Hetero resistance has a clinical implication of treatment failure and progression to a fully resistant strain. A recent study from India reported 64.8% rifampicin hetero resistance^42^. Another systematic review reported a pooled prevalence of 7% rifampicin hetero-resistance with a varying prevalence in different settings^43^. In Ethiopia, a study of clinical isolates collected through the nation-wide drug resistance survey reported low prevalence of rifampicin hetero-resistance (1.6%) among MDR-TB patients, even though the authors noted that the overall prevalence was not rare^44^. Low hetero-resistance prevalence was reported among MDR-TB strains from Pakistan (3.9%), with nearly half of the studied strains harboring compensatory mutations^45^. The high rate of rifampicin mono-resistance, accompanied by hetero-resistance, identified in this study warrants post-treatment close monitoring of EPTB for treatment failure or re-occurrence of the disease.

Evaluation of the agreement level for drug resistance detection between pDST and WGS in this study revealed a moderate (*kappa: 0.41–0.6*) to great (*kappa: 0.81–1*) agreement across a spectrum of the analyzed first and second line anti-TB drugs. There was a moderate agreement (*kappa: 0.582*) between the MTBSeq pipeline and pDST to detect rifampicin resistance. TBProfiler identified 57.1% of RR-TB detected by MTBSeq (*kappa=0.73*). As all RMR-TB were detected only through MTBSeq, the reduced agreement between the two pipelines may be explained by the incomplete repertoire of the characterized mutations in the TBProfiler databases^25^.

In this study, we observed a high rate of interim RMR-TB that remains undetected by the currently recommended critical concentration for pDST. This signifies the role of WGS in detecting interim and hetero resistances, which will aid personalized treatment strategies. Lowering the critical concentration of rifampicin from 1µg/ml to 0.5 µg/ml for pDST may benefit the detection of interim rifampicin resistance using pDST approaches^46,47^. Overall, MDR-TB, Hr-TB and interim-RMR-TB are equally important public health challenges in the realm of EPTB in Ethiopia.

This study lacks data on treatment outcomes to follow through anti-TB drug resistance patterns with clinical conditions of affected patients, which is worth addressing in future studies. While we have not characterized the protein structure of the reported compensatory mutations, further studies on the role of compensatory mutations and restored fitness in resistant strains are warranted.

## Methods

### Study design and setting

A prospective cross-sectional study was conducted on MTB isolates grown on Mycobacterium Growth Indicator Tube (MGIT) and Lowenstein Jensen (LJ) media cultured from extrapulmonary clinical specimen. The clinical samples were obtained from 542 prospectively enrolled presumptive EPTB study participants between August 2022 and October 2023(unpublished data). A total of 189 study participants were bacteriologically confirmed for EPTB. The study participants were enrolled from six high EPTB hotspot regions of central and northern part of Ethiopia, within a one-year time frame, which reflects the national representativeness of the sampling and shows a snapshot of the current EPTB status in Ethiopia. Clinical specimens were retrieved from lymph node aspirates 81% (153/189) and other clinical specimens 19% (36/189) such as pleural fluid, ascitic fluid, pericardial fluid, synovial fluid and urine. All MTB culture positive isolates were checked for purity and subjected to pDST and WGS. The study protocol was approved by the institutional ethics review boards (IRB) of the Ethiopian Public Health Institute (# EPHI-IRB-433-2022) and Addis Ababa University, College of Natural and Computational Sciences (# CNS-IRB/06/14/2022). The research was performed in accordance with the Declaration of Helsinki. Informed consent was obtained from all participants and/or their legal guardians.

### Laboratory investigations

#### Phenotypic drug sensitivity test (pDST)

Phenotypic DST was performed using BACTEC MGIT 960 at a predefined critical drug concentrations^11^ of first-line anti-TB drugs: rifampicin [RIF (1.0 µg/ml)], isoniazid [INH (0.1 µg/ml)], streptomycin [STR (1.0 µg/ml)], ethambutol [EMB (5.0 µg/ml)], and pyrazinamide [PZA (100 µg/ml)]. All isolates identified as rifampicin resistant (RR), multidrug resistant (MDR) or isoniazid resistant, rifampicin susceptible TB (Hr-TB) were further tested for second-line drug resistance at predefined critical concentrations^12^ of bedaquiline [BDQ (1 µg/ml)], clofazimine [CFZ (1 µg/ml)], delamanid [DLM (0.06 µl/ml)], linezolid [LZD (1 µg/ml), levofloxacin [(LFX (1.0 µg/ml)], moxifloxacin [MFX (0.25 µg/ml)], and ofloxacin [OFX (2 µg/ml)].

#### Nucleic acid (DNA) extraction and library preparation

MTBC isolates grown on LJ media with confluent growth of 3–4 weeks were used for sequencing. The genomic DNA extraction using the *N*-acetyl-*N*, *N*, *N*-trimethyl ammonium bromide (CTAB) method, precipitation, purification and elution was performed following the standard protocol^13^. Briefly, two to three loop-full of MTB colonies were scrubbed from LJ culture and transferred into a tube containing 400 *µ*l of Tris-EDTA (TE) buffer. The cells were heat killed with a pre-warmed heat block at 80°C for 1hr and lysed using 50 *µ*l of lysozyme at 37°C for 1h. The concentration and purity of the extracted genomic DNA was measured using the fluorometric Qubit4^14^ and spectrophotometric Nano Drop. Library preparation was done using Illumina DNA prep kit following the standard protocol^15^.

#### Whole genome sequencing (WGS) and quality control

Library preparation was performed at the Armauer Hansen Research Institute (AHRI), and whole genome sequencing was performed at the Ethiopian Public Health Institute (EPHI) using an Illumina NextSeq550 (Illumina San Diego, CA, USA) instrument.

#### WGS based drug resistance detection

To detect drug resistance associated mutations, the sequence reads were aligned to a reference genome *M. tuberculosis* H37Rv ATCC 27294 (NC_000962.3). SNPs calling was made using Sam tools v1.6^16^ at thresholds of minimum mapping quality of 20, minimum base quality at a position of 20, minimum read depth at a position of 8X, and maximum strand bias for a position of 90%. To detect hetero resistance, defined as the occurrence of mixed wild type and mutant sub population in an organism^17^, the variant calling was performed using the minimum mapping quality of 20, minimum base quality at a position of 20, minimum read depth at a position of 2X, and maximum strand bias at a position of 10%. Resistance conferring mutation was predicted using two bioinformatics pipelines, MTBseq pipeline^18^ and TB Profiler^19^, by application of the MEM algorithm of the Burrows-Wheeler alignment tool v0.7.17^20^.

#### Statistical analysis

All data were double entered into EpiData version 4.6.0.6 and exported to SPSS version 29.0 software (SPSS Inc., Chicago, Illinois, USA). Descriptive statistics and binary and multinomial logistic regression models were used to describe variables as appropriate. The probability level of <0.05 was considered statistically significant. Kappa statistics were used to evaluate the strength of agreement between pDST and WGS drug resistance prediction. We interpreted a kappa coefficient value as low agreement if (k=<0.4), moderate agreement if (k=0.41–0.6), substantial agreement if (k=0.61–0.8) and great agreement if (k=0.81–1.0), as previously described^8^.

## Figures and Tables

**Table 1:** Demographic characteristics, clinical history and phenotypic drug resistance profiles of EPTB patients, from Aug 2022-Oct 2023.

Participant characteristics	Resistance to at least one drug	Hr-TB	MDR/RR-TB	Pan susceptible	Total *n*	*p-value*
**Age**						
Mean age in years	33	40	28	32		0.158
**Sex**						
Female	8 (9.2%)	3 (3.4%)	4 (4.6%)	72 (82.8%)	87	0.905
Male	11 (10.8%)	2 (2%)	4 (3.9%)	85 (83.3%)	102	
**HIV Status**						
Reactive	1 (5.3%)	1 (5.3%)	1 (5.3%)	16 (84.2%)	19	0.350
Non-reactive	14 (9.9%)	4 (2.8%)	4 (2.8%)	119 (84.4%)	141	
Unknown	4 (13.8%)	-	3 (10.3%)	22 (75.9%)	29	
**Treatment history**						
New						
Retreatment	15 (8.8%)	3 (1.8%)	4 (2.4%)	148 (87.1%)	170	<0.001
	4 (21.1%)	2 (10.5%)	4 (21.1%)	9 (47.4%)	19	
**Contact history**						
Yes	4 (9.3%)	1 (2.3%)	4 (9.3%)	34 (79.1%)	43	0.339
No	12 (9.5%)	3 (2.4%)	3 (2.4%)	108 (85.7%)	126	
Unknown	3 (15%)		1 (5%)	15 (75%)	20	
		1 (5%)				

**Table 2:** Proportion of drug resistance among phenotypic DST and WGS-tested isolates.

Drug name	Phenotypic resistance			Genotypic resistance MTBSeq (n=160)			Genotypic resistance TBProfiler (n=160)		
	New (n=170)	Retreatment (n=19)	*p-value*	New (n=144)	Retreatment (n=16)	*p-value*	New (n=144)	Retreatment (n=16)	*p-value*
STR	4(2.4%)	6(31.6%)	<0.001	3(2.1%)	4(25%)	0.002	8(5.6%)	6(37.5%)	<0.001
INH	7(4.1%)	6(31.6%)	<0.001	5(3.5%)	5(31.3%)	<0.001	5(3.5%)	5(31.3%)	<0.001
RIF	4(2.4%)	4(21.1%)	0.024	10(6.9%)	4(25%)	0.036	4(2.8%)	4(25%)	0.004
EMB	2(1.2%)	1(5.3%)	0.275	3(2.1%)	3(18.8%)	0.014	3(2.1%)	3(18.8%)	0.014
PZA	9(5.3%)	5(26.3%)	0.007	3(2.1%)	2(12.5%)	0.079	5(3.5%)	4(25%)	0.006
CAP	6(3.6%)	1(5.3%)	0.534	-	1(1.3%)	0.100	4(2.8%)	-	0.653
KAM	4(2.4%)	1(5.3%)	0.418	-	-		-	-	-
DEL	1(0.6%)	-	1.000	-	-		1(0.7%)	-	0.9
ETO	Not tested		-	-	1(6.3%)	0.100	4(2.8%)	3(18.8%)	0.023

**Table 3:** Mutation profiles of phenotypically susceptible RMR-TB as predicted by MTBSeq pipeline.

ID	Nucleotide change	Resistant/Susceptible	Allele frequency	WHO- confidence grading
ETB_26	Ser450Ala	R	5.98	Assoc. w R-interim
**ETB_52**	**Asn438Thr**	**R**	**6.03**	**Unreported**
ETB_102	Ser450Ala	R	13.72	Assoc. w R-interim
ETB_105	Ser450Ala	R	15.63	Assoc. w R-interim
ETB_108	Ser450Ala	R	14.31	Assoc. w R-interim
ETB_130	Ser450Ala	R	19.74	Assoc. w R-interim

**Table 4:** Agreement between pDST and WGS for the detection of drug resistance and coefficient of agreement.

gDST/MTBSeq Vs. Phenotypic DST					gDST/TBProfiler Vs. Phenotypic DST			
Drug	% R within phenotypes*	% R within genotypes**	Kappa coefficient	p-value	% R within phenotypes*	% R within genotypes**	Kappa coefficient	p-value
RIF	87.5%	50%	0.582	<0.001	87.5%	87.5%	0.814	<0.001
INH	76.9%	100%	0.865	<0.001	76.9%	100%	0.865	<0.001
STR	40%	57.1%	0.414	0.003	100%	71.4%	0.784	<0.001
EMB	100%	50%	0.590	<0.001	100%	50%	0.590	<0.001
PZA	50%	100%	0.693	<0.001	80%	88.9%	0.848	<0.001

* % Phenotypically detected R also appeared resistant in gDST

** Genotypically detected R also appeared resistant in pDST

**Table 5:** List of phenotypically identified resistances with genotypic non-resistance variants

Drug	Change	Confidence grading	Type	Total-n
STR	Gly69Asp	Uncertain significance	Missense variant	3
	c.386delG	Uncertain significance	Frameshift variant	1
	c.115delC	Uncertain significance	Frameshift variant	1
	c.351delG	Uncertain significance	Frameshift variant	1
INH	Arg463Leu + Thr203Thr + Pro29Pro	Uncertain significance	Phylogenetic-SNP	2
RIF	c.−218G>A	Uncertain significance	Up-stream gene variant	1
PZA	c.390_391dupGG	Uncertain significance	Frameshift variant	1
	c.−125delC	Uncertain significance	Upstream gene variant	2
	p. Tyr41*	Uncertain significance	Stop gained	1
AMK/CAP/KAN	-187C>T	Uncertain significance	Upstream gene variant	9

**Table 6:** List of resistance conferring mutations in phenotypically susceptible new and previously treated patients

Drug	Mutation	Other pDST resistances	Confidence grading	New	Retreatment	HIV status	Total
STR	c.102delG	-	Uncertain significance	3	-	NR	3
RIF	His445Tyr + His445Pro	INH+PZA	Assoc w R-Interim	-	1	NR	1
	Ser450Ala	-	Assoc w R-Interim	5	-	NR	5
	**Asn438Thr**	-	**Unreported**	1	-	NR	1
	Val170Phe	STR+INH	Assoc w R	1	-	NR	1

EMB	Met306Val	STR+INH +RIF+PZA	Assoc w R	1	1	NR	2
	Met306Ile	+CAP	Assoc w R	1	-	Unknown	1

## Data Availability

All data associated with the main finding is provided in tables and figures. The raw sequence data generated in this study have been deposited in the National Center for Biotechnology Information (NCBI) under BioProject number PRJNA1174701.

## Abbreviations

STR: streptomycin
INH: isoniazid
RIF: rifampicin
EMB: ethambutol
PZA: pyrazinamide
AMK: amikacin
CAP: capreomycin
KAN: kanamycin
ETO: ethionamide
DEL: delamanid
